# Nanocatalysts: applications for the synthesis of N-containing five-membered heterocycles

**DOI:** 10.1039/d2ra03122a

**Published:** 2022-07-06

**Authors:** Nidhi Jangir, Surendra Kumar Bagaria, Dinesh Kumar Jangid

**Affiliations:** Department of Chemistry (Centre of Advanced Study), University of Rajasthan JLN Marg Jaipur Rajasthan 302004 India dinu.jangid@gmail.com; Department of Chemistry, Govt. Science College Sikar Rajasthan 332001 India

## Abstract

Using transition metals as nanocatalysts has opened up a vast new area in heterocyclic chemistry in the modern day. Heterocyclic moieties are significant scaffolds that have both pharmacological and industrial applications. Various scientific groups have focused their attention on the expansion of simple reaction protocols by introducing better functional group compatibilities under mild reaction conditions through the use of easily available starting materials. This review provides an outline of the applications of metallic nanoparticles as proficient, recyclable, low-cost and green heterogeneous catalysts for the preparation of a wide range of key therapeutic five-membered nitrogen-containing heterocyclic compounds as well as some other significant functionalizations over the rings. This review mainly covers the literature published through the period from 2004 to 2021.

## Abstract 2

This review article demonstrates the various synthetic pathways of five-membered nitrogen-containing heterocycles such as pyrroles, pyrazoles, imidazoles, triazoles and tetrazoles catalysed by metallic nanocatalysts.

## Graphical abstract

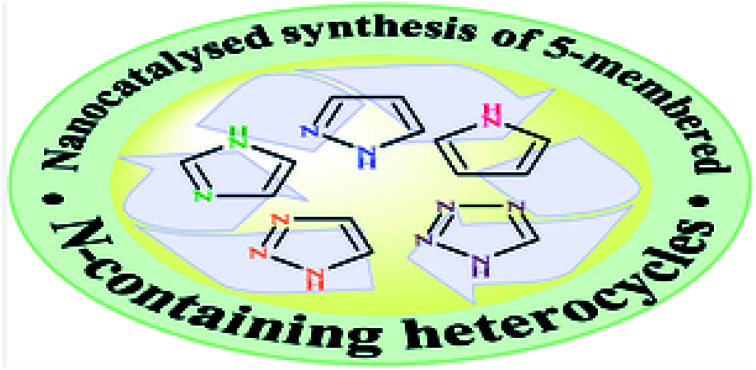

## 1. Introduction

Heterocycles are not only vital because of their extensive presence in organic chemistry but are also very significant due to their chemical, biological and industrial applications. Various compounds such as anti-oxidant, anti-inflammatory, anti-bacterial, anti-viral and anti-tumoral agents,^1–5^ alkaloids, hormones, essential amino acids, vitamins, hemoglobin, a range of dyes and man-made drugs comprise heterocycles as core scaffolds. These heterocycles are also present in corrosion inhibitors, herbicides, agrochemicals and other materials. Heterocycles also act as chief components in organic synthesis, are an important part of drug molecules and possess industrial applications in cosmetics, reprography, plastics and information storage.^6–10^ The development of heterocyclic compounds has been known to comprise reactions such as cascade reactions, cyclization and annulation. Carbocycle, heterocycle and complex natural product formation utilize the catalyst-supported method. With the help of catalysts, procedures like synthesis and retrosynthesis become very easy and simple.^11–13^

**Fig. 1:**
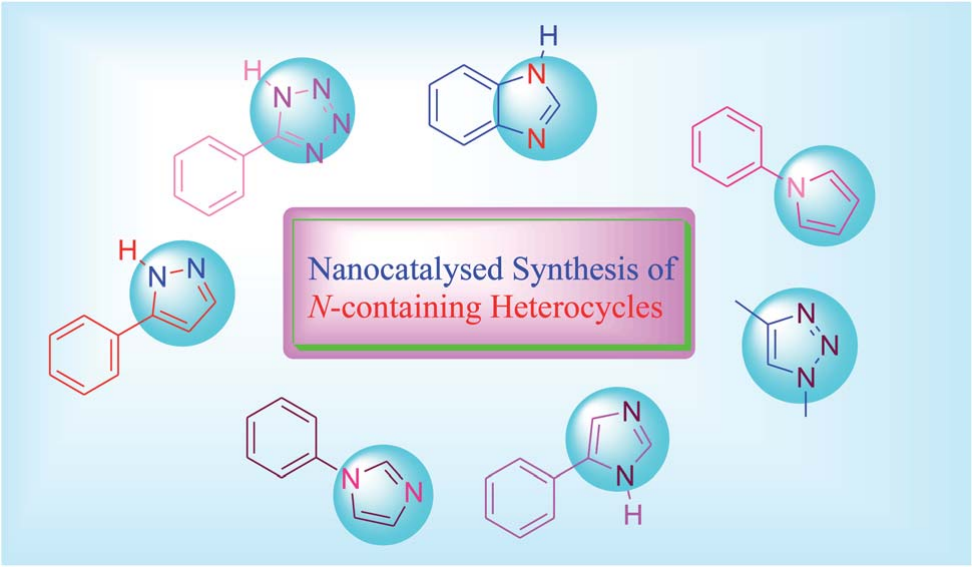
Five-membered heterocycles.

Nitrogen-containing heterocycles are incredibly useful in the fields of pure and applied chemistry. N-Heterocycles are used as therapeutic compounds and form the base for numerous drugs such as morphine (analgesic), vincristine (cancer chemotherapy) and captopril (hypertension).^14,15^ Five-membered N-heterocyclic compounds are especially significant in numerous applications and are found in a variety of drugs and natural products.^16,17^ Aromatic five-membered N-heterocycles having one to four nitrogen atoms comprise pyrroles, pyrazoles, imidazoles, 1,2,3-triazoles, 1,2,4-triazoles and tetrazoles. Five-membered nitrogen-containing heterocycles such as indoles, pyrroles and carbazoles occur in a number of bioactive active compounds (Fig. 1). Due to their various applications, synthetic chemists show constant attention to the functionalization and development of these heterocyclic compounds. Saturated five-membered N-heterocycles are not only important for the formation of drugs, pigments and pharmaceuticals but are also used for the progress of organic functional materials.^18^ Thus, the synthesis of these compounds has brought about long-lasting attention. Several procedures for the preparation of these heterocyclic compounds include carbon–nitrogen bond-forming reactions such as reductive amination, nucleophilic substitution and dipolar cycloaddition for ring closure.^19–21^

A number of procedures have been developed for the formation of these compounds, which involve the use of ultrasound irradiation,^22,23^ catalysts,^24,25^ and microwave irradiation.^26,27^ These procedures have their individual advantages but they also possess certain limitations such as complex instruments, inaccessible materials, non-selectivity, and non-recyclability. To conquer these problems, the use of nanocatalysts holds great potential.^28^ Although previous work has been done on N-containing five-membered heterocycles (pyrrole, pyrazoles, imidazole, triazole, and tetrazole)^29^ and published by scientists individually, we have combined those reviews into a single review that will be helpful for the scientific community.

This review covers the development of the nano-catalysed synthesis of the following five-membered nitrogen-containing heterocycles:

Nano-catalysed synthesis of pyrrole.

Nano-catalysed synthesis of pyrazole.

Nano-catalysed synthesis of imidazole.

Nano-catalysed synthesis of triazole.

Nano-catalysed synthesis of tetrazole.

## 2. Nano-catalysed synthesis of pyrrole

**Scheme 1:**
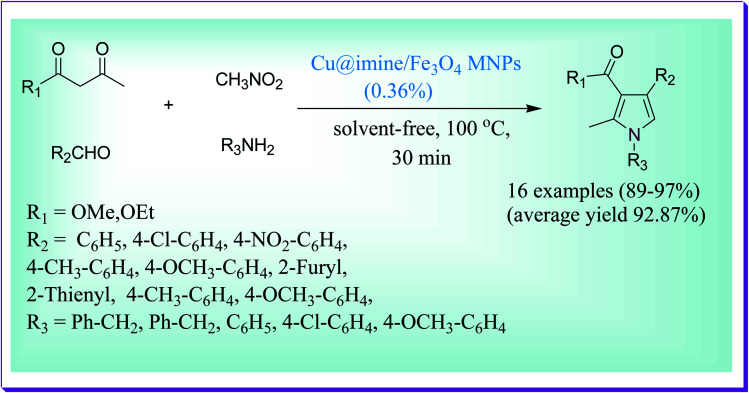
Cu@imine/Fe_3_O_4_ MNP-catalysed formation of polysubstituted pyrrole derivatives.

An effective method was reported by Thwin *et al.* for the quick preparation of bioactive polysubstituted pyrrole derivatives through a four-component reaction between ethyl acetoacetate, nitromethane, benzaldehyde and aniline under solvent-free conditions and in a short reaction time by virtue of the Cu@imine/Fe_3_O_4_ MNP catalyst. The presented method demonstrates several key features such as low catalyst loading, simple isolation of the product, low reaction times, excellent yields, cleansing of the products, and high recoverability and reusability of the catalyst. The used catalyst was also reused five to six times without any substantial loss in its catalytic efficiency (Scheme 1).^30^

**Scheme 2:**
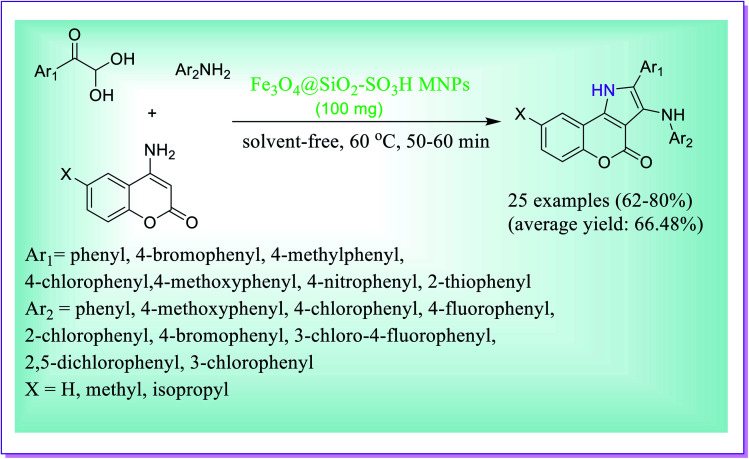
Fe_3_O_4_@SiO_2_–SO_3_H-catalysed preparation of chromeno[4,3-*b*]pyrrol-4(1*H*)-one derivatives.

A simple and green one-pot method was reported by Mukherjee *et al.* for the preparation of bioactive chromeno[4,3-*b*]pyrrol-4(1*H*)-one derivatives under solvent-free conditions *via* the three-component domino condensation reaction of 4-aminocoumarins, arylglyoxal monohydrates and arylamines catalysed by Fe_3_O_4_@SiO_2_–SO_3_H nanoparticles as a solid acid catalyst. Prominent characteristics of the current protocol are low reaction times, mild reaction conditions, exclusion of risky solvents, excellent yield of the desired products and the use of a magnetically isolable and reusable nanocatalyst (Scheme 2).^31^

**Scheme 3:**
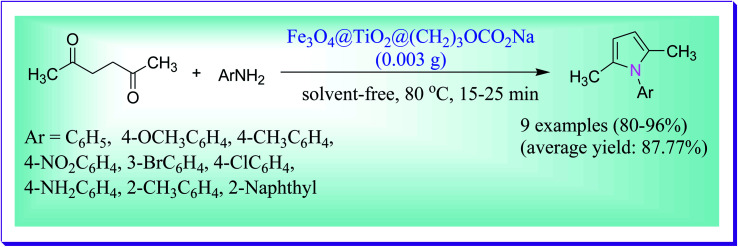
Preparation of pyrrole derivatives catalysed by Fe_3_O_4_@TiO_2_@(CH_2_)_3_OCO_2_Na.

Fattahi *et al.* reported a novel Paal–Knorr reaction under green and mild conditions for the preparation of pyrrole derivatives catalysed by the novel Fe_3_O_4_@TiO_2_-supported sodium carbonate (Fe_3_O_4_@TiO_2_@(CH_2_)_3_OCO_2_Na) catalyst. Moreover, it is observed that for the preparation of pyrroles through the Paal–Knorr reaction, this base-functionalized magnetic catalyst shows superb activity. The isolation of this catalyst is completed in a simplistic way using an external magnetic field and it can be reused up to five times without any loss in its catalytic activity. Some of the key features of the presented protocol are mild reaction conditions, catalyst recyclability, excellent yield of the product, low reaction times and long storage (Scheme 3).^32^

**Scheme 4:**
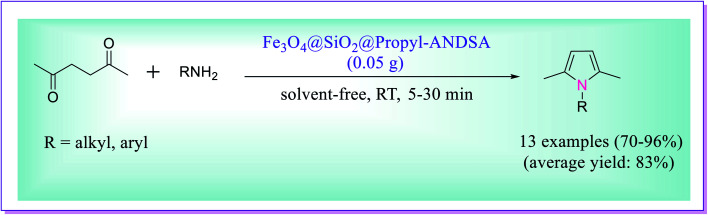
Preparation of substituted pyrroles in the presence of Fe_3_O_4_@SiO_2_@propyl–ANDSA magnetic nanoparticles (MNPs).

Ghorbani *et al.* developed an efficient protocol for the preparation of *N*-substituted pyrroles by the reaction of primary amines (1 mmol) and 2,5-hexanedione (1 mmol) catalysed by Fe_3_O_4_@SiO_2_@propyl–ANDSA MNPs. For the preparation of *N*-substituted pyrroles, the reaction was investigated at different temperatures in a variety of solvents such as H_2_O, EtOH, H_2_O/EtOH, and CH_3_CN, as well as under solvent-free conditions, and the finest result was obtained at room temperature in CH_3_CN and using 0.05 g of the catalyst (Scheme 4). The catalyst has the potential of revival and reusability for five to seven cycles without any significant loss in its activity.^33^

**Scheme 5:**
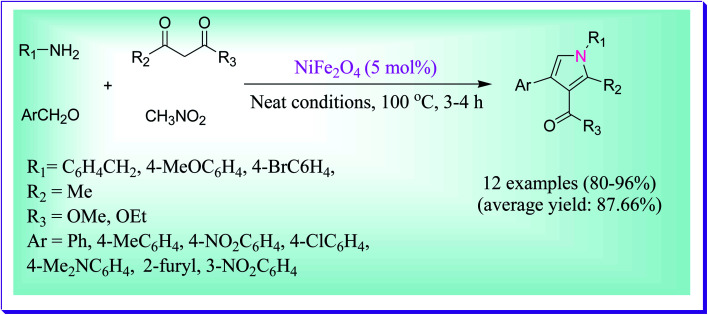
Preparation of substituted pyrrole derivatives catalysed by nickel ferrite nanoparticles.

An efficient protocol was reported by Moghaddam *et al.* for the preparation of a wide range of substituted pyrroles using nickel ferrite nanoparticles, a proficient and reusable nanomagnetic catalyst. If the nickel ferrite nanocatalyst was not used, low amount of the optimized product was observed. Under neat conditions, the one-pot, four-component reaction of aldehydes, amines, 1,3-dicarbonyl compounds and nitromethane was carried out at 100 °C for 3–4 h. Notably, the nickel ferrite nanocatalyst was magnetically isolated and reused for five cycles without any significant loss in its catalytic activity (Scheme 5).^34^

**Scheme 6:**
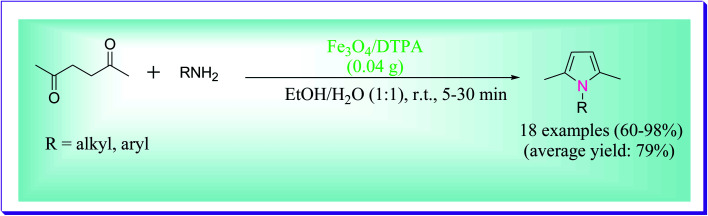
Preparation of *N*-substituted pyrroles catalysed by Fe_3_O_4_/DTPA.

The protocol reported by Hemmati *et al.* involved the synthesis of *N*-substituted pyrroles through the reaction between primary amines (1 mmol) and 2,5-hexanedione (1 mmol) in the catalytic presence of Fe_3_O_4_/DTPA. For the preparation of *N*-substituted pyrroles, the reaction was tested in the presence of various amounts of catalyst in diverse solvents such as CCl_4_, CH_3_CN, CH_2_Cl_2_, H_2_O, EtOH, and EtOH/H_2_O (1 : 1) as well as solvent-free conditions, through which the best results were obtained at room temperature using 0.04 g of the catalyst in EtOH/H_2_O (1 : 1) (Scheme 6).^35^

**Scheme 7:**
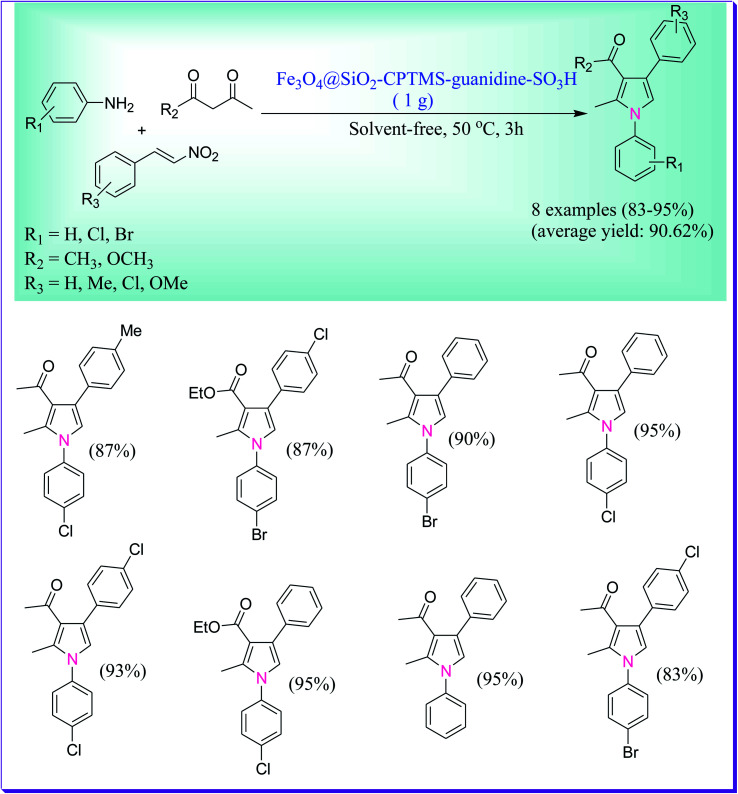
Fe_3_O_4_@SiO_2_–CPTMS–guanidine–SO_3_H-catalysed synthesis of polysubstituted pyrroles.

An efficient nanocatalytic protocol was developed by Rostami *et al.* for the one-pot preparation of polysubstituted pyrrole derivatives by the reaction of aromatic amines, β-diketones or β-ketoesters, and β-nitrostyrene in the nanocatalytic presence of Fe_3_O_4_@SiO_2_–CPTMS–guanidine–SO_3_H under solvent-free conditions.^36^ The main advantages of the current process over the earlier reported ones are its low reaction times, solvent-free conditions, low reaction temperature, the use of a nanocatalyst that is easily extracted from the reaction mixture and excellent yields (Scheme 7).

**Scheme 8:**
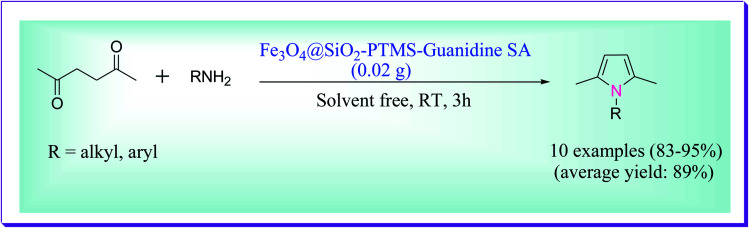
Preparation of *N*-substituted pyrroles catalysed by Fe_3_O_4_@SiO_2_–PTMS–guanidine–SA magnetic nanoparticles (MNPs).

Shiri *et al.* demonstrated the efficient synthesis of *N*-substituted pyrroles by the reaction of primary amines (0.5 mmol) and 2,5-hexanedione (0.5 mmol) catalysed by Fe_3_O_4_@SiO_2_–PTMS–guanidine–SA MNPs. For the preparation of *N*-substituted pyrroles, the reaction was tested in the presence of different amounts of the catalyst in a variety of solvents such as H_2_O, PEG, DMF, EtOH, CH_3_CN, and toluene as well as solvent-free conditions, in which the best result was obtained at room temperature using 0.02 g of the catalyst and under solvent-free conditions (Scheme 8). The used catalyst was reused for five to six consecutive cycles without any substantial loss in its activity.^37^

**Scheme 9:**
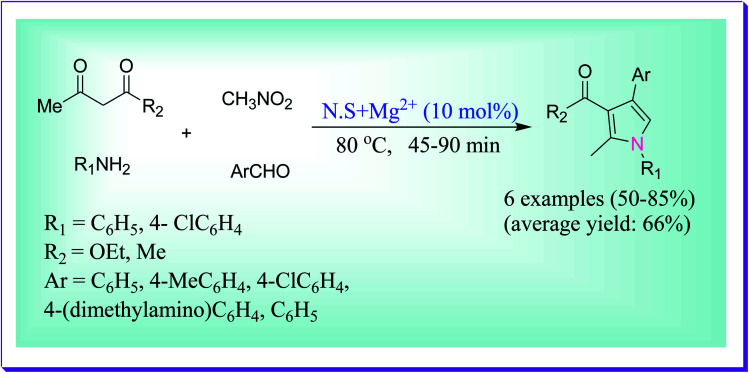
Synthesis of polysubstituted pyrroles under solvent-free conditions.

Fakhree *et al.* demonstrated the proficient solvent-free synthesis of 1,2,3,4-tetrasubstituted pyrrole derivatives using green magnesium-integrated white sandstone as a novel heterogeneous catalyst through the four component one-pot reaction of 1,3-dicarbonyl compounds, aromatic aldehydes, aniline derivatives and nitromethane.^38^ Their work revealed that a superior yield of the desired product was obtained when the catalyst loading was 10% w/w at 80 °C (Scheme 9). The activity of the catalyst did not decrease for up to five successive reactions.

**Scheme 10:**
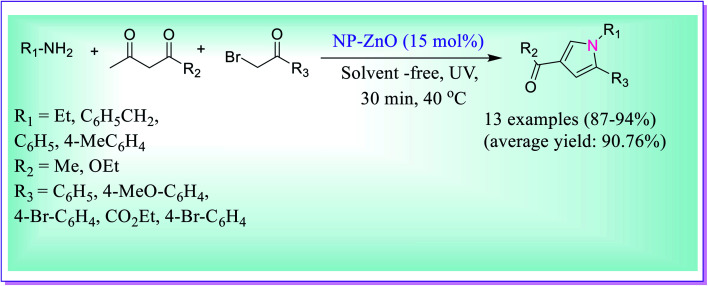
Preparation of tetrasubstituted pyrrole derivatives catalysed by ZnO nanoparticles.

An efficient solvent-free protocol for the preparation of 1,2,3,5-tetrasubstituted pyrrole derivatives was developed by Shahvelayati *et al. via* three-component one-pot formation using amines, β-dicarbonyls and α-bromoketones under sonochemical using ZnO nanoparticles (Scheme 10).^39^ The desired product was obtained in good yields using the highly capable nano ZnO particles. The recyclability and reusability of the catalyst and solvent-free conditions make this reaction highly noteworthy.

## 3. Nano-catalysed synthesis of pyrazole

**Scheme 11:**
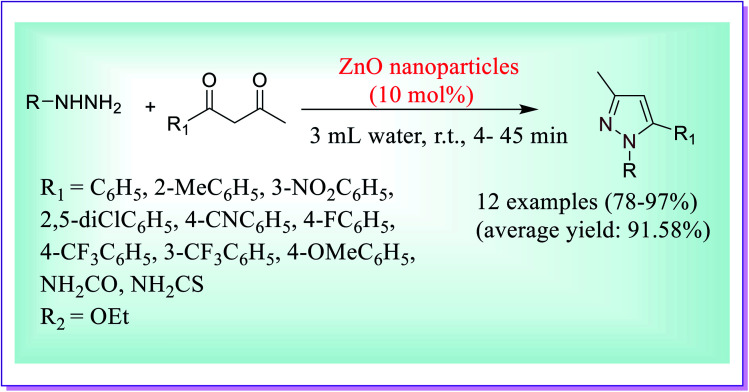
ZnO-catalysed preparation of 3-methyl-1-phenyl-1*H*-pyrazol-5-ol.

For the preparation of 3-methyl-1-phenyl-1*H*-pyrazol-5-ol, an efficient, concise, quick and environmentally benign protocol was developed by Girish *et al.* by the reactions of phenyl hydrazine (1 mmol) and ethyl acetoacetate (1 mmol) at room temperature in water using bulk ZnO (5 mol%) for about 25 minutes (Scheme 11). In the absence of ZnO NPs, the required product was obtained in low quantity; even after using 10 mol% of the catalyst and increasing the reaction time (90 min), there was no considerable change in the product yield. However, when the reaction was carried out using ZnO NPs (5 mol%) as the catalyst at room temperature, it was observed that after 25 minutes, the required product was obtained in moderate quantity and on further increase in the molar ratio of the catalyst, the required product was obtained in good yield and low reaction time.^40^

Thus, this process for the formation of substituted pyrazole derivatives includes several key advantages such as its speed, easy work-up procedure, avoidance of hazardous organic solvents, good to high yields and eco-friendly acceptability. The nano ZnO catalyst was reused for subsequent reactions for five to six runs without any degradation in its catalytic activity.

**Scheme 12:**
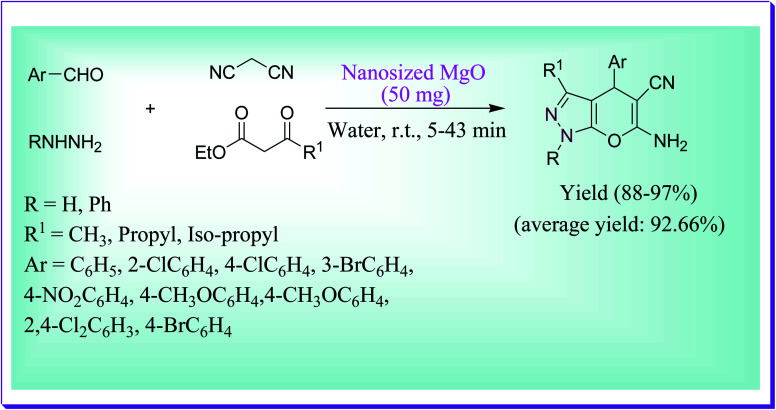
MgO-catalysed preparation of dihydropyrano[2,3-*c*]pyrazole derivatives.

Nanosized magnesium oxide (MgO) could be used as a nanocatalyst for the proficient preparation of dihydropyrano[2,3-*c*]-pyrazole derivatives by the reaction of substituted hydrazine, substituted aldehydes, malononitrile, and 3-oxopropanoate, as reported by Babaie *et al.* (Scheme 12). The total quantity of MgO nanocatalyst used is 50 mg in water for an appropriate reaction time at room temperature (RT), and an excellent amount of the required products was obtained.^41^

It was also observed for the current protocol that the prepared MgO nanocatalyst showed higher catalytic activity in terms of yield and reaction time in comparison with commercially available MgO. The authors also reported the effect of substituents on the yield and found that superb yields were obtained with electron-donating and withdrawing substituents on the aromatic aldehydes.

**Scheme 13:**
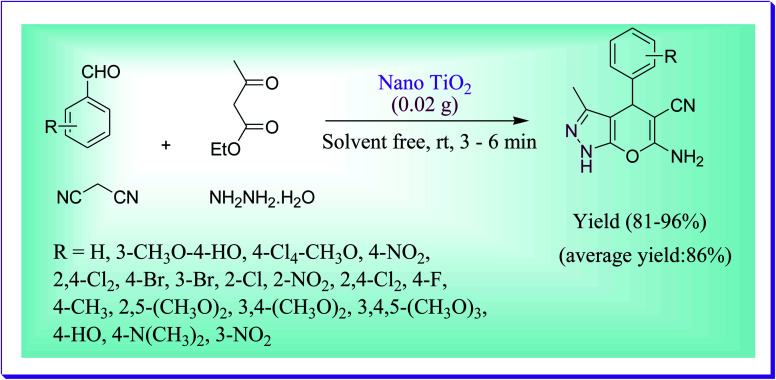
TiO_2_-catalysed development of pyrano[2,3-*c*]-pyrazole derivatives.

Titanium dioxide was developed as a novel nanocatalyst for the synthesis of dihydropyrano-[2,3-*c*]-pyrazoles *via* a one-step route by Reza *et al.*^42^ The corresponding product was obtained by the condensation reaction of the substituted aldehydes, hydrazine hydrate, malononitrile, and ethyl acetoacetate under solvent-free conditions at RT. The excellent yield of product could be obtained when 0.25 mmol of nano-titania was used. The respective mechanism of this reaction involved a Knoevenagel condensation between malononitrile and aldehyde form to yield 2-benzylidenemalononitrile as an intermediate, and after that Michael addition, followed by intramolecular cyclisation with the intermediate, yielded the dihydropyrano-[2,3-*c*]-pyrazoles (Scheme 13).

**Scheme 14:**
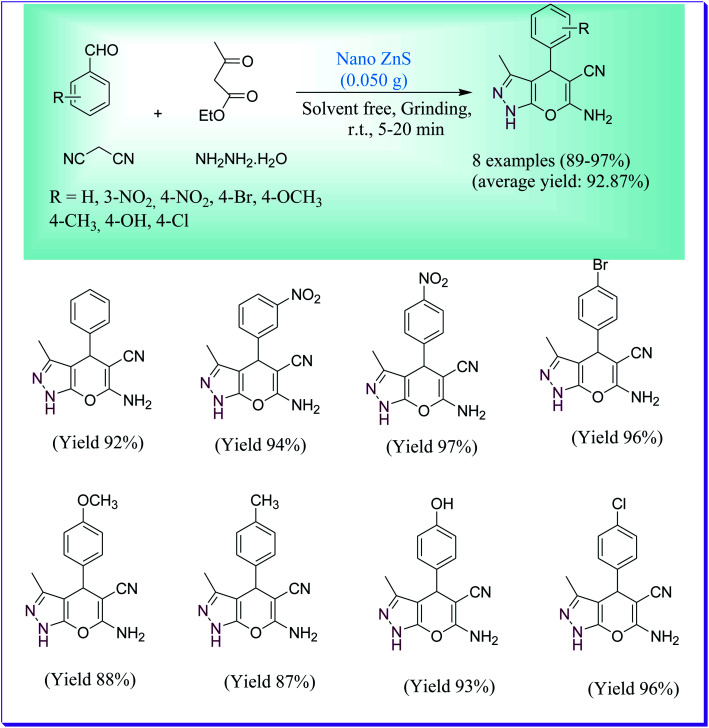
Synthesis of pyranopyrazole derivatives using the ZnS nanocatalyst.

ZnS nanoparticles designed *via* a hydrothermal method were used as a nanocatalyst for the synthesis of pyrano[2,3-*c*]-pyrazoles reported by Borhade *et al.*^43^ The four-component condensation reaction between malononitrile, aromatic aldehydes, hydrazine hydrate and ethyl acetoacetate under solvent-free media by the grinding technique was effectively achieved by use of ZnS nanoparticles and the product was formed in superior yields in 12 min (Scheme 14). The used nanocatalyst could be isolated by simple filtration and could be reused for up to five consecutive steps without any degradation in catalytic properties. The attentive properties of this protocol are green solvent-free reaction conditions and low catalyst requirement.

**Scheme 15:**
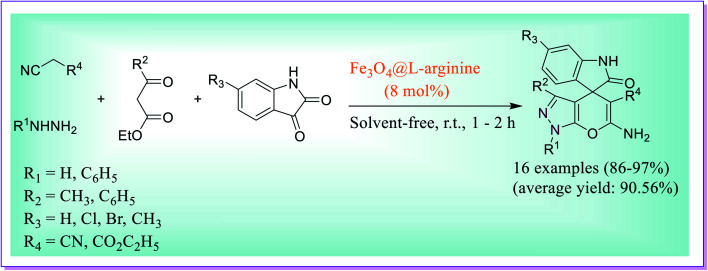
Fe_3_O_4_@L-arginine-catalysed preparation of spiro-pyrano[2,3-*c*]-pyrazole.

A one-pot novel protocol was developed for the preparation of spiro-pyrano[2,3-*c*]-pyrazole using Fe_3_O_4_@l-arginine as a heterogeneous catalyst by Ghasemzadeh *et al.*^44^ The reaction involved four-component condensation between hydrazine derivatives, substituted isatin, β-keto esters, and dynamic methylene compound under solvent-free reaction conditions. The best yield of the product was obtained when 8 mol% of the nanocatalyst was used at RT with 60 min of reaction time. The catalytic activity of the nanocatalyst does not lose significantly up to five steps because it was observed that the product yield was not decreased in successive steps (Scheme 15).

**Scheme 16:**
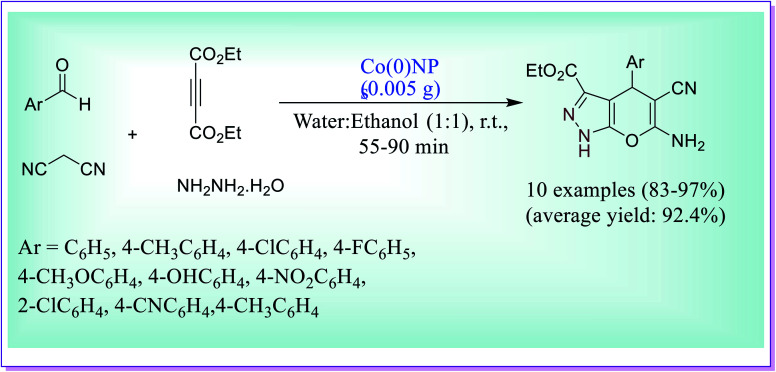
CoNP-catalysed synthesis of pyranopyrazole.

Cobalt nanoparticles (Co NPs) which were prepared using an aqueous extract of *Zingiber* could be used as a heterogeneous catalyst for the preparation of pyrano-[2,3-*c*]-pyrazole. The product *i.e.* pyrano-[2,3-*c*]-pyrazole was obtained by the condensation reaction of substituted aldehydes, hydrazine hydrate, malononitrile and diethyl acetylenedicarboxylate (DAD) under aqueous media of ethanol.^45^ 0.005 g of nanocatalyst and 1 h reaction time at room temperature are the best conditions to achieve the product in excellent amount. Easy workup procedure, mild reaction conditions, and high yield of product were the useful advantages of the used protocol (Scheme 16).

**Scheme 17:**
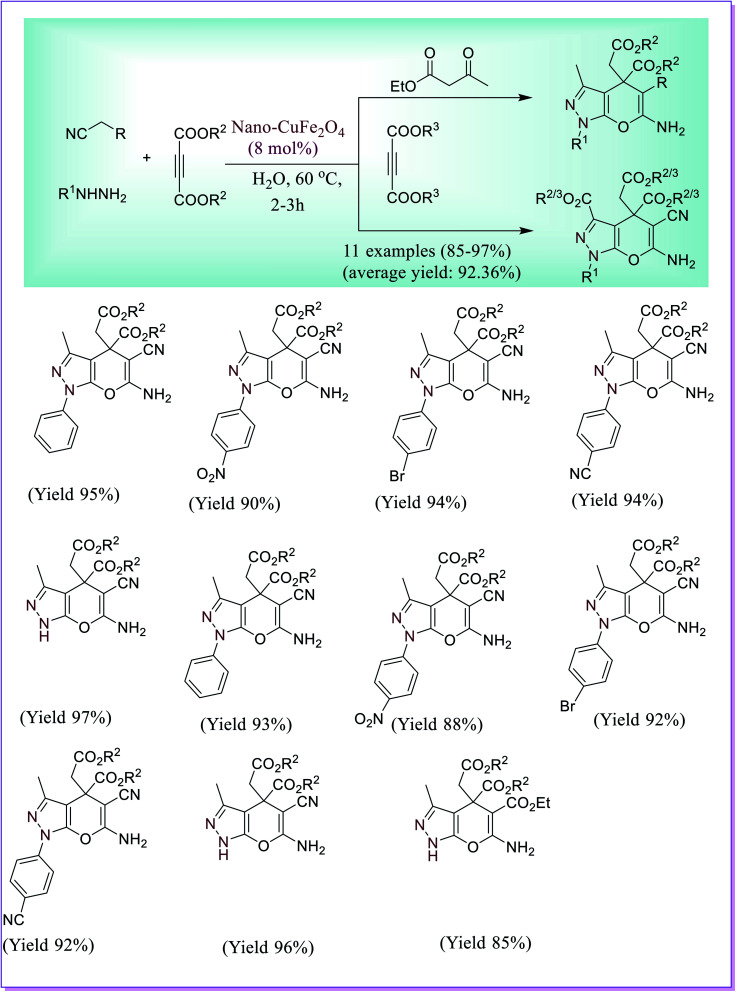
Copper ferrite-catalysed preparation of pyrano[2,3-*c*]pyrazole.

An efficient nanocatalyst, copper ferrite (CuFe_2_O_4_), was synthesised by Pradhan *et al.*^46^ using the citric acid complex method. The copper ferrite catalyst was used efficiently for the formation of pyrano[2,3-*c*]-pyrazoles by four-component reaction of malononitrile or ethyl cyanoacetate, hydrazine hydrate, dialkyl acetylenedicarboxylate and ethyl acetoacetate. The reliable mechanism involved a Knoevenagel type condensation between hydrazine and ethyl acetoacetate, and in a further step, a Michael type addition occurred between malononitrile and diethyl acetylenedicarboxylate (DAD). After that Fe^3+^ Lewis acidic sites and Cu^2+^ active sites catalyse the above intramolecular cyclization of two intermediates. This novel procedure offered many benefits, like highly functional group tolerance, simple workup, eco-friendly reaction conditions and significant yield of the corresponding products (Scheme 17).

**Scheme 18:**
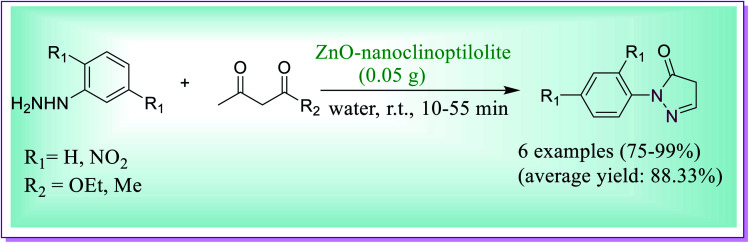
ZnO–nanoclinoptilolite catalysed synthesis of pyrazole derivatives in water.

Kakhki *et al.* described an efficient water phase protocol for the synthesis of pyrazole derivatives through the reaction of arylhydrazines and β-dicarbonyl compounds catalysed by ZnO–nanoclinoptilolite. The presented protocol was investigated in different solvents (H_2_O, dimethyl formamide (DMF) or ethanol). But the study revealed that by using DMF and EtOH, lower yield of the product (68 or 69) was obtained in longer reaction time in comparison to water as a solvent (Scheme 18).^47^

**Scheme 19:**
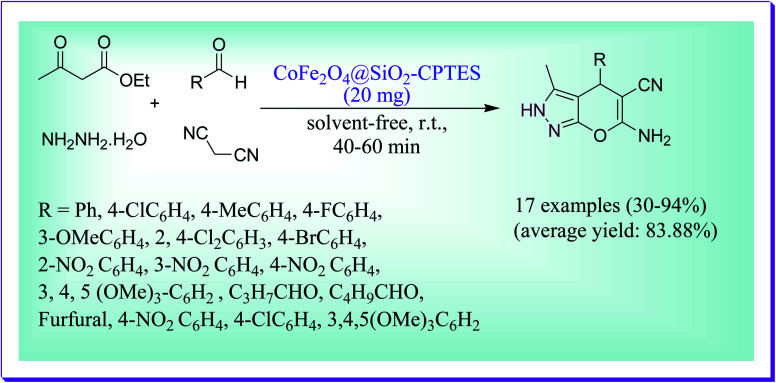
Nanocomposite-catalysed synthesis of pyranopyrazole derivatives.

Dadaei *et al.* documented an expedient and solvent-free green way for the preparation of 6-amino-2,4-dihydropyrano[2,3-*c*]pyrazol-5-carbonitrile derivatives by virtue of the CoFe_2_O_4_ silica supported containing melamine as a magnetic heterogeneous nanocatalyst through the reaction of different aldehydes, ethyl acetoacetate, hydrazine hydrate, and malononitrile in 1 : 1 : 1 : 1 mole ratio at room temperature (Scheme 19). The used catalyst CoFe_2_O_4_@SiO_2_–CPTES–melamine is green, simply recoverable and reusable several times without any lack in its catalytic efficiency.^48^

**Scheme 20:**
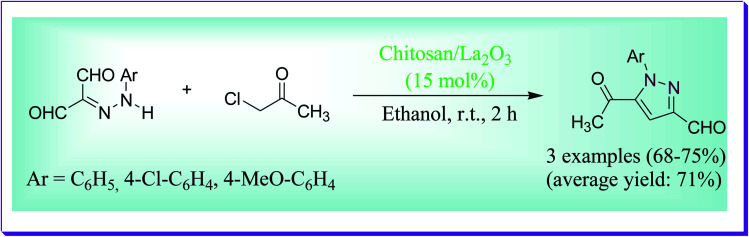
Synthesis of 5-acetyl-1-aryl-1*H*-pyrazole-3-carbaldehyde.

Khalil *et al.* have discussed high yielding preparation of 5-acetyl-1-aryl-1*H*-pyrazole-3-carbaldehyde under refluxing conditions through the reaction of 2-arylhydrazone-malono-1,3-dial and chloroacetone by virtue of chitosan/La_2_O_3_ nanocomposite proceeding efficiently to afford the consequent pyrazoles in good yield (Scheme 20). Over, the results revealed that for the reaction chitosan/La_2_O_3_ is better in acting capably as a basic promoter due to its synergistic effect and also due to its simplicity of recovery and recycling.^49^

## 4. Nano-catalysed synthesis of imidazole

**Scheme 21:**
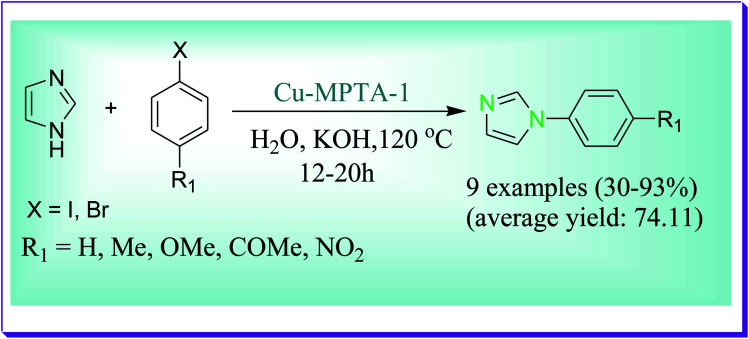
*N*-Arylation of imidazoles with different aryl halides.

A model *N*-arylation reaction described by Salam *et al.* was conducted to analyse the applicability of the copper catalyst to N–H heterocycles with diverse aryl halides and imidazole in water as a solvent by using KOH at 120 °C.^50^ For the one-pot coupling reaction the Cu-nanocatalyst which has been formed by grafting of Cu(ii) at the surface of mesoporous polymer MPTA-1 has been effectively used as a heterogeneous catalyst for the *N*-arylation reaction of imidazole with aryl halides in water as a green solvent. The desired product was formed in good to excellent yields. Thus, this catalytic procedure offers a number of advantages like easy work-up process, being economical, being eco-friendly and reusability of the catalyst (Scheme 21).

**Scheme 22:**
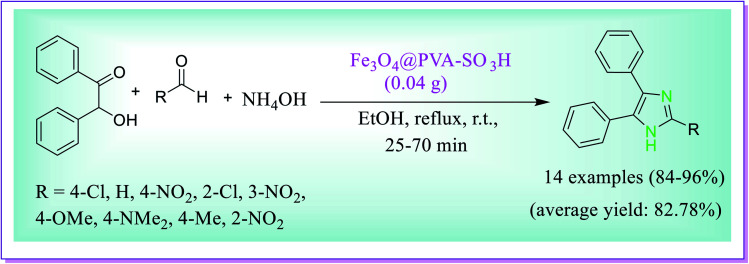
Synthesis of 2,4,5-trisubstituted imidazole catalysed by the Fe_3_O_4_@PVA–SO_3_H nanocatalyst.

This protocol developed by Maleki *et al.* is a highly efficient, facile, green approach for the preparation of substituted imidazole by using a recoverable novel magnetic Fe_3_O_4_@PVA–SO_3_H nanocatalyst. This methodology involves the condensation reactions of benzyl or benzoin, aldehydes and ammonium acetate in refluxing EtOH in the presence of magnetite nanoparticles for the synthesis of excellent yields of trisubstituted imidazole in suitable time under mild reaction conditions. The better results were achieved when the reaction was carried out in the presence of 0.04 g catalyst for 40 min in refluxing EtOH. After the completion of reaction, the catalyst was simply separated using a magnet and the solid product so obtained was purified by recrystallization from ethanol (Scheme 22).^51^

**Scheme 23:**
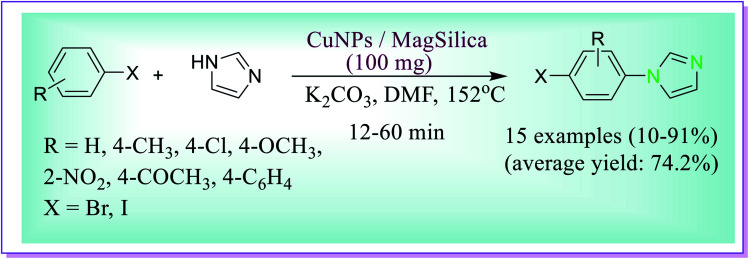
Formation of 1-phenyl-1*H*-imidazoles catalysed by Cu NPs/MagSilica.

Nador *et al.* developed the formation of 1-phenyl-1*H*-imidazole by the reactions of bromobenzene (1.0 mmol) and imidazole (1.0 mmol) catalysed by the copper nanoparticles dispersed on silica-coated maghemite (CuNPs/MagSilica) catalyst (100 mg, 11 mol% Cu), in DMF as the solvent and K_2_CO_3_ (2.0 mmol) as the base. After 24 h, the desired product 1-phenyl-1*H*-imidazole was formed in 52% yield. But if the base is not used the yield decreases to 4%; therefore, the existence of a base is a requisite for the reaction to occur. So, a novel methodology was fruitfully developed for the *N*-(hetero)arylation of imidazole with (hetero)aryl bromides and iodides under ligand-free conditions which was catalysed by a magnetically recoverable nanocatalyst consisting of copper nanoparticles on nanosized silica-coated maghemite. High atom economy, simple work-up procedure, and reuse of the catalyst several times make this methodology a very attractive alternative for the synthesis of *N*-(hetero)aryl imidazole (Scheme 23).^52^

**Scheme 24:**
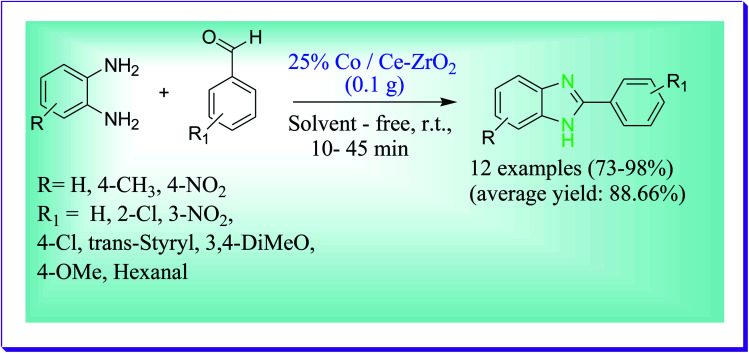
Synthesis of 2-substituted-1*H*-benzimidazoles catalysed by Co/Ce–ZrO_2_.

This protocol developed by Behbahani *et al.* described the condensation reaction of *o*-phenylenediamine (1 mmol) with 3-nitro benzaldehyde (1 mmol) for the preparation of 2-substituted benzimidazoles catalysed by 25% Co/Ce–ZrO_2_ devoid of solvent for 15 min at room temperature. It was observed that the best results were obtained by 25% Co/Ce–ZrO_2_ nano fine particles and if the 25% Co/Ce–ZrO_2_ nano fine particles were not used then the required product was obtained in less yield. So the catalyst plays a vital role in the synthesis of 2-substituted benzimidazoles. It was shown that at room temperature when benzaldehyde (2 mmol) in the presence of *o*-phenylenediamine (1 mmol) was stirred, the mixture of 1,2-disubstituted and 2-substituted benzimidazole derivatives was formed in the ratio 70 : 30. When the outcome of solvent was examined, it was observed that solvent-free stands to be of best choice because of its quick reaction rate, excellent yield, cheapness and environmental acceptability.^53^ This protocol has many advantages like less reaction time, high yields of the products, ecological and green aspects, avoiding unsafe solvents and use of recyclable nanocatalysts (Scheme 24).

**Scheme 25:**
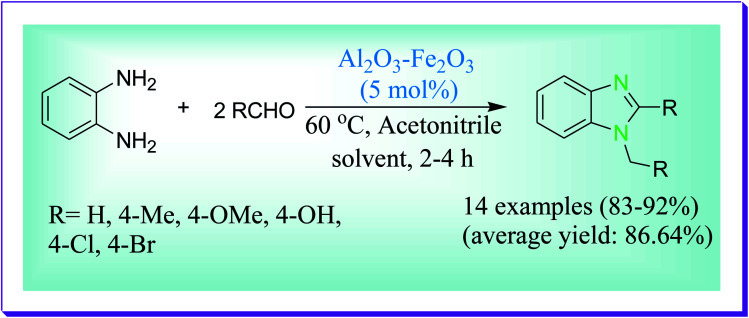
Al_2_O_3_–Fe_2_O_3_-catalysed preparation of 1,2-disubstituted benzimidazoles.

Kaushik *et al.* have demonstrated a green protocol for the preparation of 1,2-disubstituted benzimidazoles^54^ under mild reaction conditions in acetonitrile catalysed by Al_2_O_3_–Fe_2_O_3_ heterogeneous nanocrystals which have high surface area and high catalytic activity (Scheme 25). In the presented protocol the used nanocatalyst Al_2_O_3_–Fe_2_O_3_ is environmentally benign, recyclable and reusable and there is no use of unsafe solvents.

**Scheme 26:**
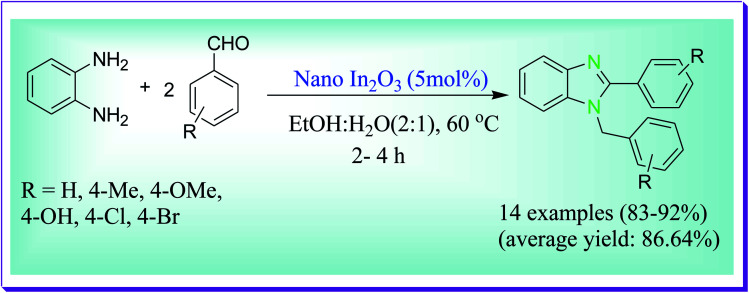
Preparation of 1,2-disubstituted benzimidazoles catalysed by nano In_2_O_3_.

Hajra *et al.* reported the formation of 1,2-disubstituted benzimidazoles *via* the condensation of diamine and aldehydes by virtue of nano In_2_O_3_ catalyst in aqueous media under mild reaction conditions. This protocol is appropriate for aryl, aliphatic and heteroaryl aldehydes. Common applicability, working ease, mild reaction conditions, and aqueous reaction media are the noteworthy importance of the current process. In_2_O_3_ nanoparticles can be easily recyclable and reusable (after the third run, recovery amount, 90% and yield, 82%), devoid of any loss in its considerable catalytic activity (Scheme 26).^55^

**Scheme 27:**
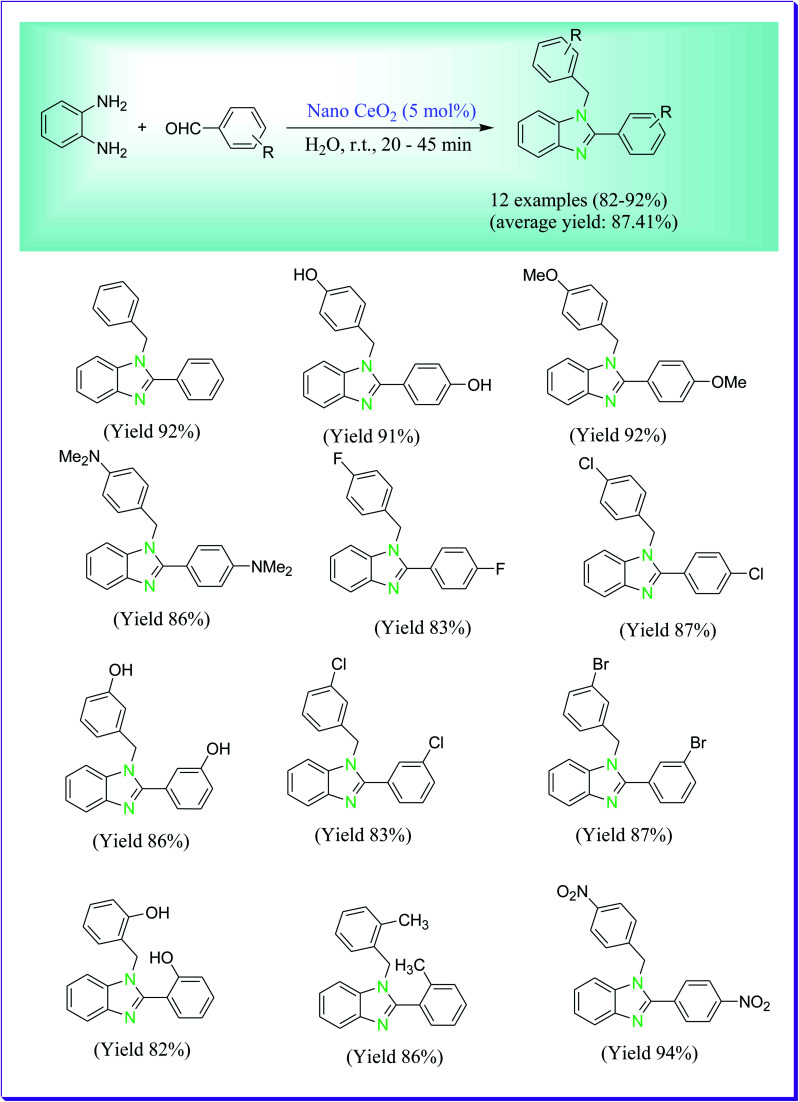
Nano ceria-catalysed preparation of 1,2-disubstituted benzimidazoles.

A proficient, choosy, economical and green route was described by Shelkar *et al.* for the preparation of 1,2-disubstituted benzimidazoles from the reaction of 1,2-phenylenediamine with 2 mol of benzaldehyde catalysed by nano ceria (CeO_2_) as a proficient heterogeneous catalyst at room temperature with water as a solvent which is the significant consideration of an eco-friendly path of preparation in organic chemistry.^56^ In the case of catalyst concentration, a better yield of products was obtained by using 5 mol% of CeO_2_. In comparison to aliphatic counter parts, better yield of the product was obtained by aromatic aldehydes. The heterogeneous catalyst which is used is simply distinguishable and recyclable up to three successive cycles, lacking any degradation in its catalytic property (Scheme 27).

**Scheme 28:**
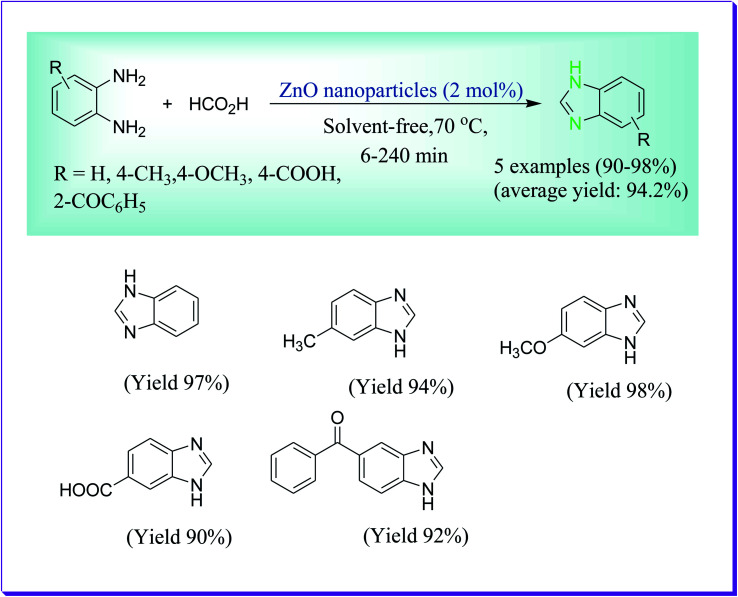
Formation of benzimidazole derivatives catalysed by NAP-ZnO.

This protocol reported by Alinezhad *et al.* described the cyclo-condensation reaction of *o*-phenylenediamines and carboxylic acids or their derivatives in excellent yields. For the formation of benzimidazole and its derivatives, a number of processes have been published in the literature, for the reason of their extensive scope in pharmacological activity, industrial and synthetic applications, while many of the earlier reported protocols have limitations such as harsh reaction conditions, side reactions, less yields, high reaction temperature, lengthy reaction time and requisite of costly reagents and contaminated solvents. Consequently, the invention of a mild and practical path for synthesis of derivatives of benzimidazole continues to draw the interest of researchers (Scheme 28).^57^

**Scheme 29:**
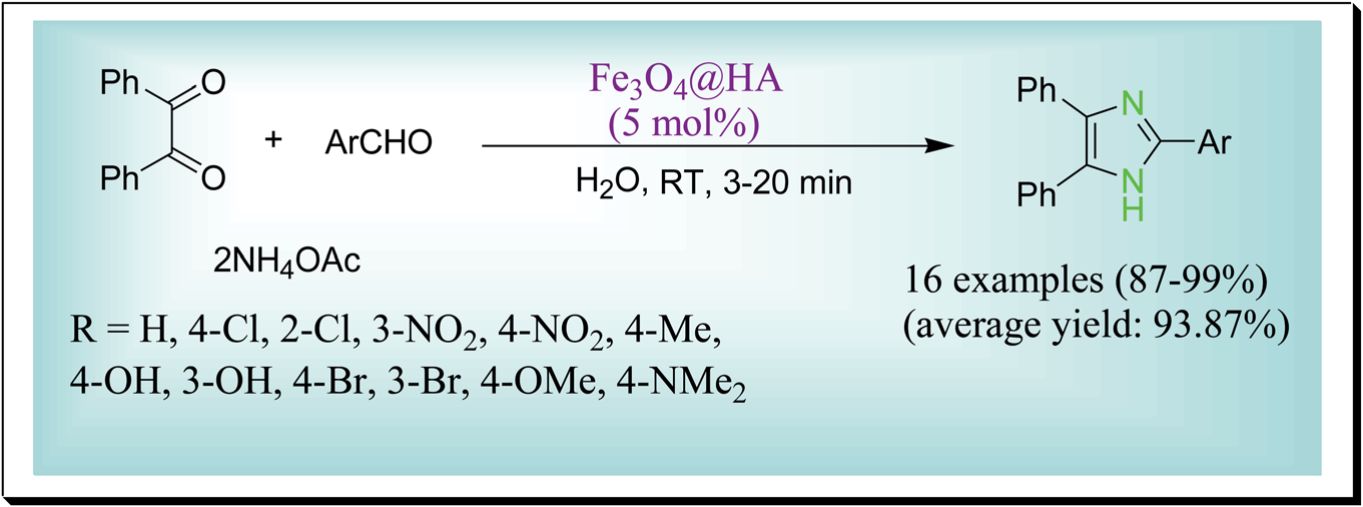
Preparation of trisubstituted imidazoles catalysed by Fe_3_O_4_@HA nanoparticles.

Hosseini *et al.* have disclosed an efficient one-pot preparation of 2,4,5-trisubstituted imidazoles catalysed by a magnetically solid acid nanocatalyst Fe_3_O_4_@HA at room temperature in water (Scheme 29). By this process, the desired products are obtained in high to excellent yields and in suitable times. Moreover, by a magnetic field, this nanocatalyst can be easily recovered and recycled up to six successive reaction runs without any obvious reduction in its catalytic efficiency. The key features of the used nanocatalyst are being proficient, magnetic isolation, reusability and eco-friendly catalyst with a natural source.^58^

**Scheme 30:**
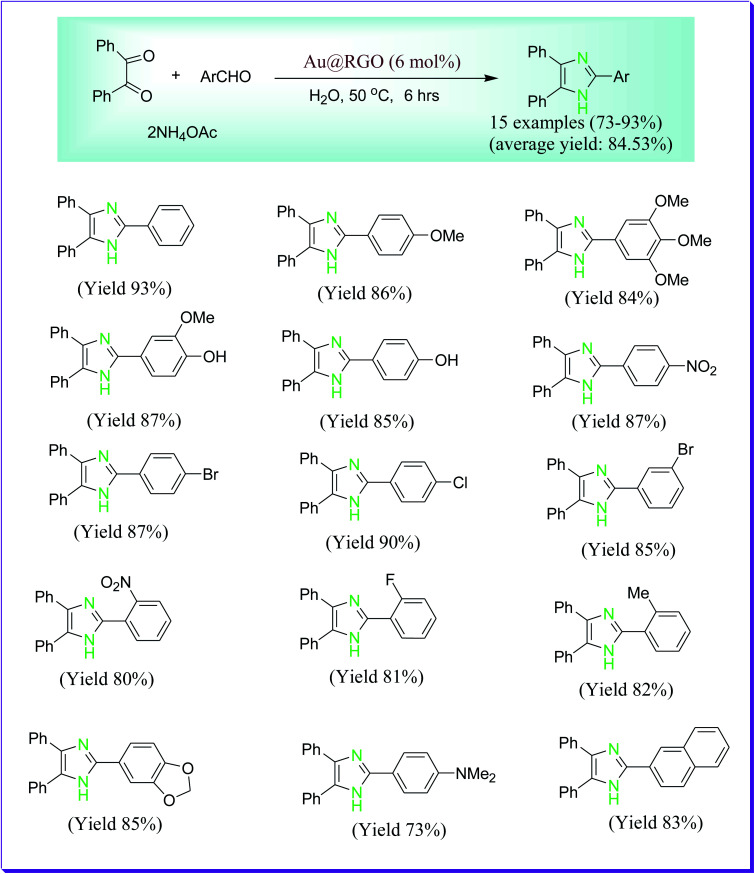
Preparation of 2,4,5-trisubstituted imidazoles catalysed by graphene oxide-supported gold nanocatalyst.

Sinha *et al.* have demonstrated the preparation of 2,4,5-trisubstituted imidazoles by virtue of a graphene oxide supported gold nanocatalyst in aqueous media (Scheme 30). The key features of the presented route are being eco-compatible, operational simplicity, reusability of the catalyst and water as a green solvent. The used catalyst can be easily recovered for the successive reactions and reused for several runs, lacking any considerable decrease in its catalytic activity.^59^

## 5. Nano-catalysed synthesis of triazole

**Scheme 31:**
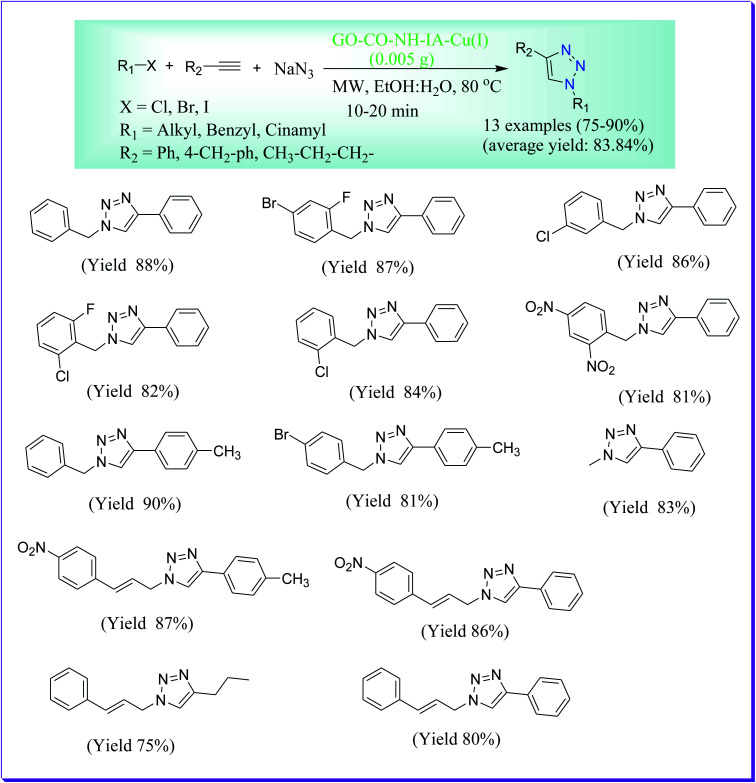
GO–NH–1A-Cu(i)-catalysed preparation of 1,4-disubstituted 1,2,3-triazoles.

Naeimi *et al.* have discussed a proficient, economical, concise and quick protocol for the preparation of 1,4-disubstituted 1,2,3-triazoles *via* the three-component reaction of alkyl azide, alkyl halide and sodium azide. After that, alkyl azide undergoes 1,3-dipolarcycloaddition reaction with terminal alkynes catalysed by GO–NH–1A–Cu(i) under microwave irradiation to form the required product in excellent yields and in short reaction times. This procedure has several advantages like reusability of catalyst, stability, simple work-up procedure, inexpensive in nature and green conditions. This process can be useful for the formation of a broad range of 1,2,3-triazoles to yield the required product in high to superb yields (Scheme 31).^60^

**Scheme 32:**
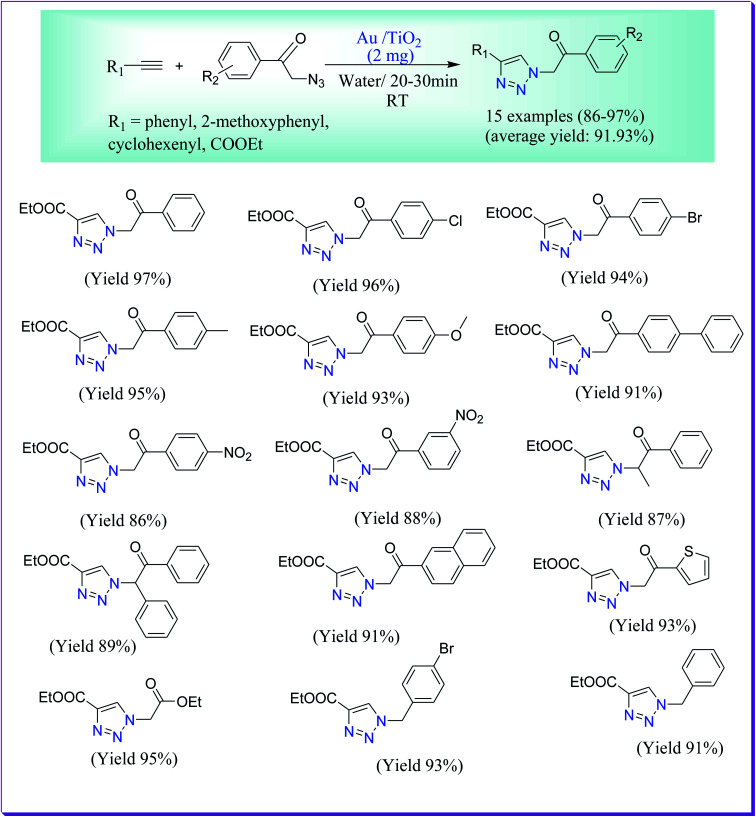
Titania-supported Au NP-catalysed 1,2,3-triazole preparation.

Boominathan *et al.* have successfully prepared the 1,4-disubstituted 1,2,3-triazole derivative in moderate to excellent yield as the sole product^61^ by Huisgen [3+2] cycloaddition of azides with a variety of terminal alkynes in aqueous medium by virtue of titania-supported AuNPs. The NPs were formed by the deposition–precipitation method. For the foremost period it is reported that Au/TiO_2_ can be used as a dynamic catalyst in the Huisgen [3+2] cycloaddition of azides with terminal alkynes in water (Scheme 32).

**Scheme 33:**
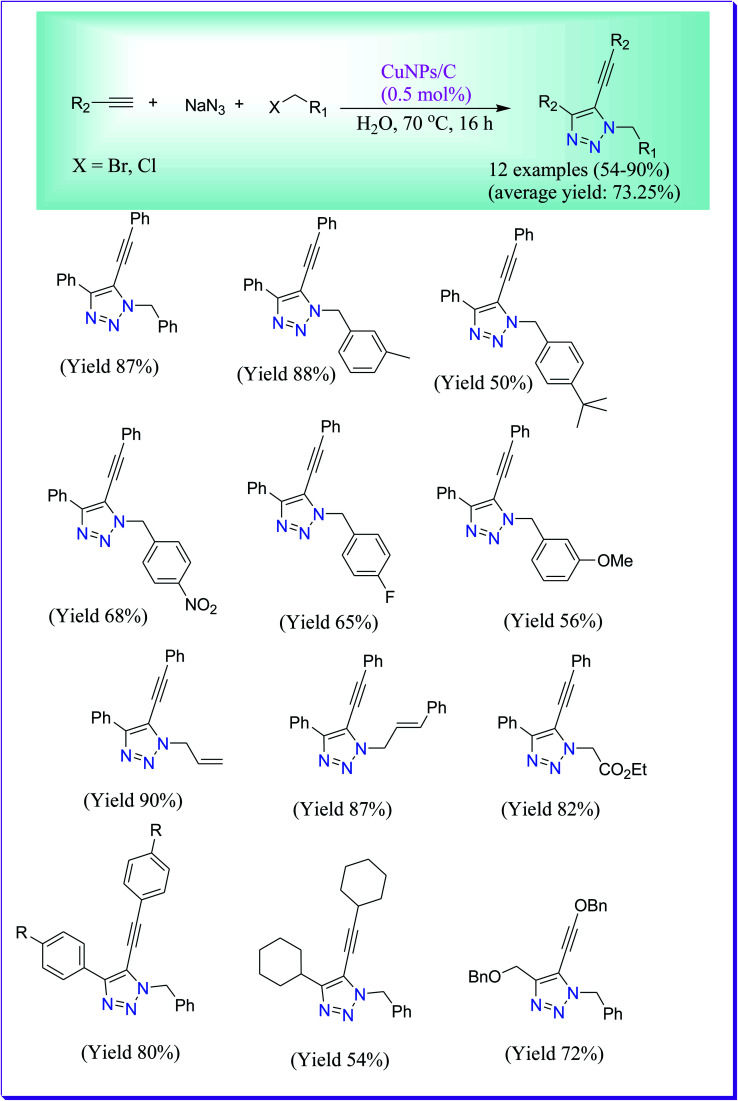
CuNPs/C-catalysed preparation of 1,2,3-triazole.

Alonso *et al.* have discussed high yielding synthesis of β-hydroxytriazoles through the multicomponent click reaction of propargyl methyl ether in water catalysed by CuNPs/C at 70 °C to form the corresponding desired product.^62^ The catalyst is simple to form, reusable at a low copper loading (0.5 mol%), and shows superior catalytic activity in comparison to other commercially accessible copper sources. In addition, the catalyst could be reused, with insignificant leaching and recycled four times, yielding moderate to excellent amount of the required product (Scheme 33).

**Scheme 34:**
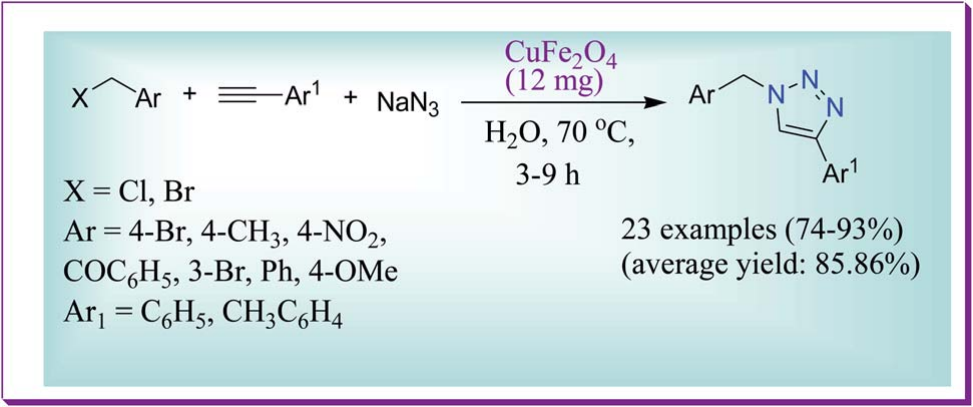
Preparation of the organic azide.

Kumar *et al.* discussed high yielding one-pot formation of 1,2,3-triazoles. This synthesis of 1,2,3-triazoles involved preliminary substitution of benzyl halides to sodium azide to produce *in situ* benzyl azides which is followed by copper ferrite catalysed cycloaddition reaction with alkynes in water at 70 °C. Electron donating substituents like methyl and methoxy and electron withdrawing substituents such as bromo and nitro groups at the *para* position of benzyl bromide were uniformly effectual toward the nucleophilic substitution of azide, followed by 1,3-dipolar cycloaddition.^63^ The process mentioned here is easy, simplistic and can be appropriate to a broad variety of substrates with high functional group tolerance (Scheme 34).

**Scheme 35:**
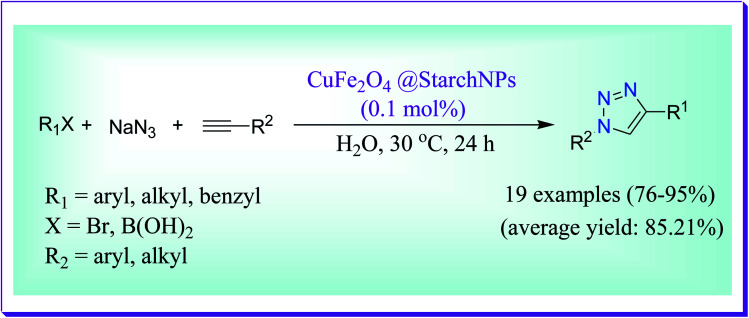
Application of CuFe_2_O_4_ nanoparticles in the ‘click’ reaction.

An efficient protocol reported by Bonyasi *et al.* to shows the appliance of CuFe_2_O_4_@starch nanoparticles for ‘click’ synthesis of 1,2,3-triazoles in aqueous medium. By the use of this magnetically separable heterogeneous catalyst a variety of alkyl halides, benzylic halides, alkyl bromides and arylboronic acids reacted effectively with sodium azide and alkynes to form 1,4-disubstituted 1,2,3-triazoles in excellent yields (Scheme 35).^64^

**Scheme 36:**
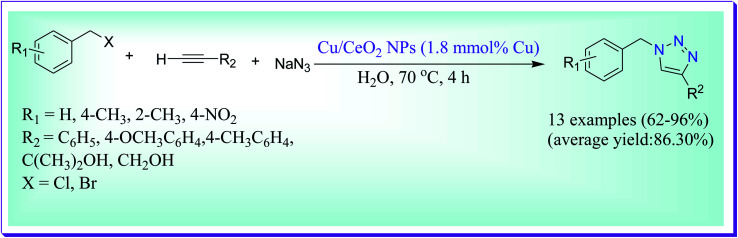
CuNP/CeO_2_-catalysed preparation of 1,4-disubstituted-1,2,3-triazoles.

Amini *et al.* have demonstrated a green method for the preparation of 1,2,3-triazoles in good yields catalysed by CuNPs/CeO_2_*via* the 1,3-dipolar cycloaddition of terminal alkynes with organic azides generated *in situ* from sodium azide and diverse organic halides in water at 70 °C.^65^ The prominent characteristics of the current procedure are less reaction time, mild reaction conditions, simple operation, reusability of the catalyst, eagerly accessible preliminary materials and reagents and applicability to a wide range of substrates (Scheme 36).

**Scheme 37:**
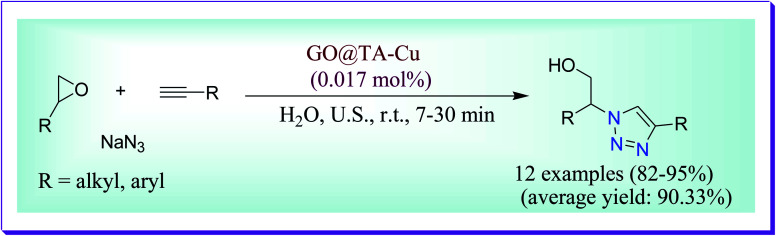
Synthesis of β-thiol-1,2,3-triazoles catalysed by the GO@polytriazole–Cu nanocatalyst.

Naeimi *et al.* have demonstrated efficient one-pot synthesis of β-thiol-1,4-disubstituted-1,2,3-triazoles in aqueous medium by the reaction of sodium azide, terminal alkynes, and various oxiranes catalysed by GO@polytriazole–Cu nanocomposite. The current procedure has numerous advantages like less reaction times, excellent yields of product, ideal regioselectivity, use of the eco-friendly solvent, easy work-up procedure, reusability and simple isolation of the heterogeneous magnetic catalyst. The recycled catalyst was used for many cycles without any deterioration in its catalytic activity (Scheme 37).^66^

**Scheme 38:**
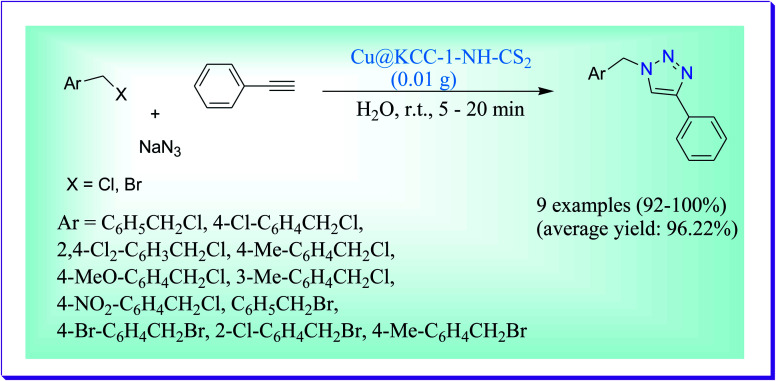
Synthesis of 1,4-disubstituted 1,2,3-triazoles in aqueous medium.

Anvari *et al.* have discussed highly efficient one-pot synthesis of 1,4-disubstituted 1,2,3-triazoles in aqueous medium with a green solvent by virtue of Cu@KCC-1–NH–CS_2_ a green nanocatalyst at 50 °C by the reaction of bromo derivatives, alkynes and azide in excellent yields in very less time (Scheme 38). The advantages of the current protocol are high surface area, permeable structure of the nanocatalyst, high catalytic presentation and green solvent with less reaction times (5–20 min), easy work up procedure, no use of toxic solvents, and appropriate reusability of the catalyst. The used nanocatalyst can easily be isolated with uncomplicated filtration and again used with lack of reduction in its catalytic performance.^67^

**Scheme 39:**
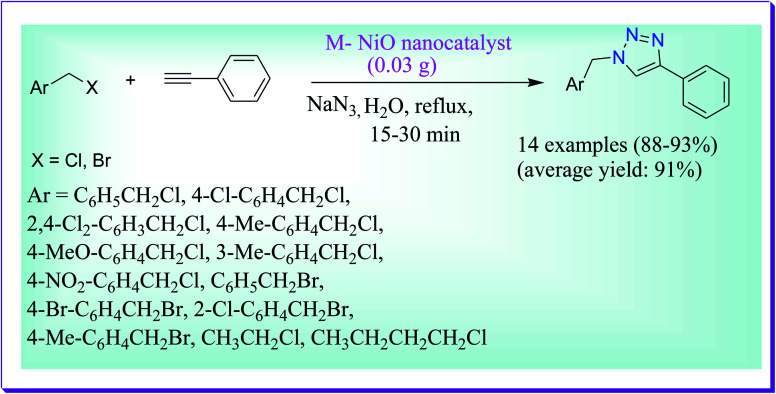
M–NiO nanocatalyst-catalysed synthesis of triazole derivatives.

Hashemi *et al.* have developed a simple and efficient protocol for the regioselective synthesis of 1,4-disubstituted-1,2,3-triazoles catalysed by melamine-supported nickel oxide nanoparticles (M–NiO nanocatalyst) in aqueous medium (Scheme 39). In this route, there is no use of supplementary substances such as reducing reagents. Some of the advantages of the presented route are excellent yields of products, simple work-up and clean procedure. Furthermore, the catalyst can be easily isolated and reused at least 6 times without any noteworthy reduction in its catalytic efficiency.^68^

**Scheme 40:**
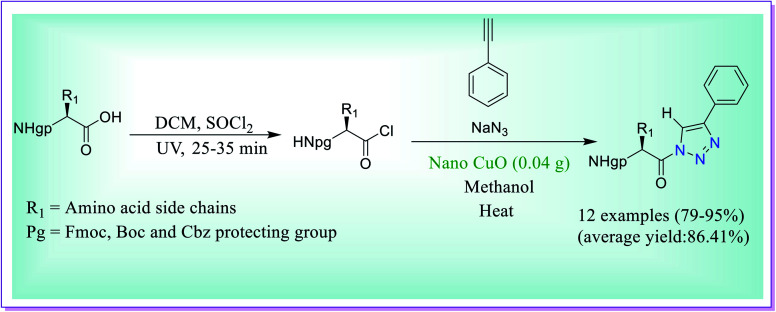
Nano-CuO-catalysed preparation of *N*^α^-protected amino keto-1,2,3-triazoles.

Raghavendra *et al.* have disclosed preparation of *N*^α^-protected amino keto-1,2,3-triazoles through the three-component reaction between amino acyl chloride derivatives of protected amino acids/dipeptide acids, phenylacetylene and sodium azide catalysed by the nano-CuO catalyst (Scheme 40). Many of the procedures for the formation of 1,2,3-triazoles reported in the literature suffer from the problems like use of huge surplus costly chemicals, inconsiderate situation, prolonged process, low yields and several *in situ* generated intermediates which need unique concern for the final products. The presented procedure is secure, well-organized for the production of titled heterocyclic compounds, and the process does not need separation of the acyl azide intermediates.^69^

## 6. Nano-catalysed synthesis of tetrazoles

**Scheme 41:**
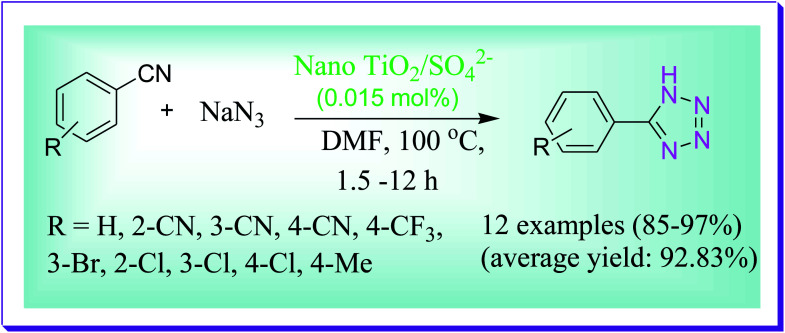
Nano TiO_2_/SO_4_^2−^-catalysed preparation of 5-substituted-1*H*-tetrazoles.

Herein a simple, eco-friendly, efficient protocol was developed by Hosseini-Sarvari *et al.* for the synthesis of tetrazoles by the reactions of benzonitrile and sodium azide by using environmentally benign nano TiO_2_/SO_4_^2−^ as a catalyst to yield 5-phenyl-1*H*-tetrazoles in excellent yields. On screening a diversity of structurally divergent benzonitriles and generality of the nano sulphated titania promoted [3+2] cycloaddition reaction to yield 5-substituted 1*H*-tetrazoles, it was observed that aromatic benzonitriles give moderate to good yields and the superior results were achieved by nitriles having an electron withdrawing group at the *para*- or *meta* position in comparison to the electron donating substituents.^70^ This protocol has several key features such as simple work-up, straightforward methodology, excellent yield of the desired product, simple formation and handling of the catalyst; this catalyst can be re-obtained by filtration and reused for substituent reactions six to seven times, devoid of any loss in the catalytic properties. This methodology has extensive use in organic synthesis for the formation of 5-substituted-1*H*-tetrazoles (Scheme 41).

**Scheme 42:**
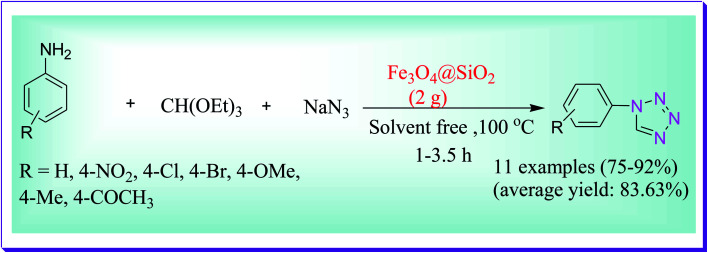
Preparation of 1- and 5-substituted 1*H*-tetrazoles.

Dehghani *et al.* reported the formation of 1- and 5-substituted 1*H*-tetrazoles through nitriles and amines by virtue of magnetite nanoparticle immobilized salen Cu(ii) as a reusable catalyst.^71^ This nanocatalyst can be simply isolated by using a magnetic field and can be reused for consequent seven runs without any loss in the catalytic activity (Scheme 42).

**Scheme 43:**
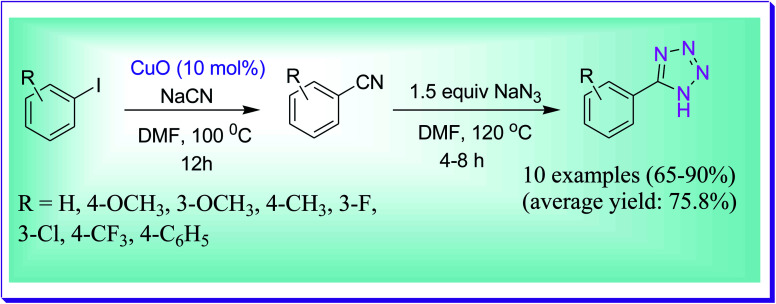
Copper(ii) oxide NP-catalysed preparation of 5-substituted 1*H*-tetrazoles.

Yapuri *et al.* developed synthesis of the cyanation of aryl iodides to the consequent aryl nitriles by virtue of copper(ii) oxide nanoparticles as a catalyst and sodium cyanide as a cyanide source. The obtained aryl nitriles consequently transformed into 5-substituted 1*H*-tetrazoles *via* one-pot [2+3] cycloaddition catalysed by copper and by using a sodium azide source (Scheme 43).^72^

**Scheme 44:**
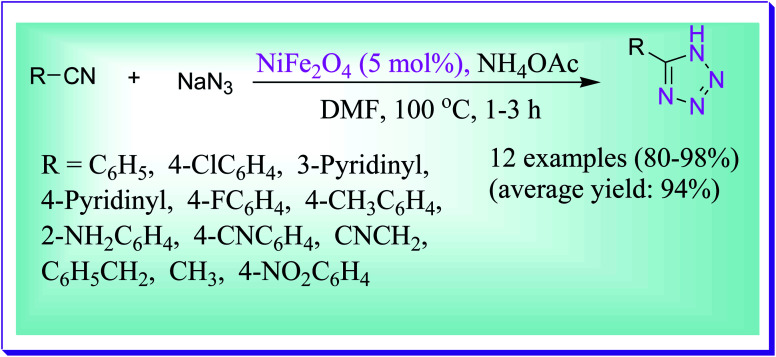
NiFe_2_O_4_-catalysed preparation of tetrazole derivatives in DMF.

Abrishami *et al.* reported the synthesis of 5-substituted 1*H*-tetrazoles, *via* simple one-pot [2+3] cycloaddition of sodium azide to a variety of nitriles in DMF by virtue of magnetically recoverable nickel ferrite nanoparticles as a catalyst at 100 °C for 13 h. This model reaction is unsuccessful in the absence of the nickel ferrite nanocatalyst even after a lengthy period of time. Even after five runs, there is no noteworthy reduction in the catalytic activity in this protocol (Scheme 44).^73^

**Scheme 45:**
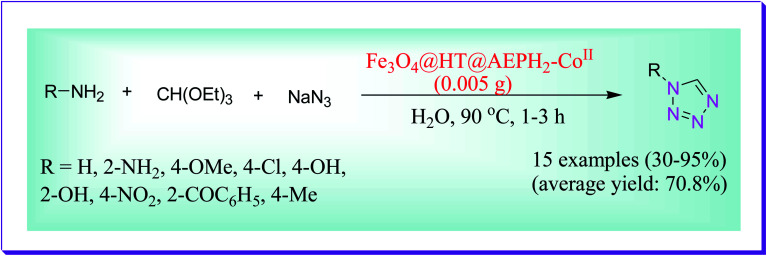
Fe_3_O_4_@HT@AEPH_2_–Co^II^-catalysed preparation of tetrazole derivatives.

An efficient protocol was investigated by Salimi *et al.* for the preparation of 1-substituted-1*H* tetrazole derivatives from the condensation reaction of sodium azide, triethyl orthoformate and diversity of heterocyclic/aromatic amines catalysed by an environmentally benign heterogeneous catalyst (Fe_3_O_4_@HT@AEPH_2_–Co ii) and H_2_O was used as the solvent at 90 °C. This protocol includes several benefits such as catalyst stability and recyclability, solvent-free conditions, easy procedure yield of products, least chemical wastes, no toxicity, short reaction times, recyclability, purity and less reaction time.^74^ Recuperation of the catalyst can be simply run out by an external magnet by the reaction medium and the catalyst could be reused 6 times, devoid of any noteworthy deterioration in catalytic activity. The planned procedure could be useful to a broad scale of the substrate (*i.e.*, electron-deficient and electron-rich) (Scheme 45).

**Scheme 46:**
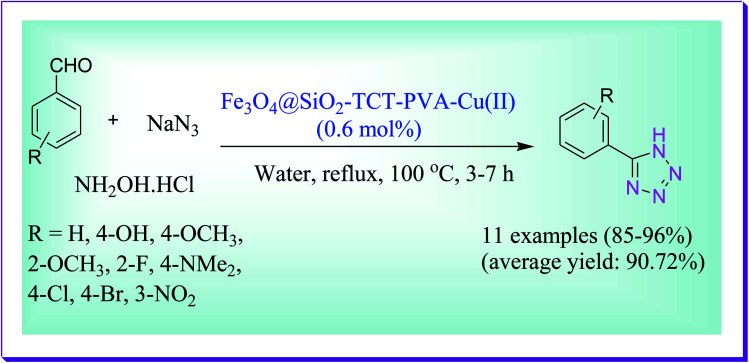
Fe_3_O_4_@SiO_2_-TCT–PVA–Cu(ii)-catalysed synthesis of 5-substituted 1*H*-tetrazoles.

Sardarian *et al.* have disclosed the one-pot three-component reactions accomplished through the click reaction at refluxing water in the presence of Fe_3_O_4_@SiO_2_–TCT–PVA–Cu(ii) NPs to form the 5-substituted 1*H*-tetrazoles with high yields by the reaction of aldehydes, hydroxylamine hydrochloride and sodium azide devoid of using external ligands or additives as promoters. The catalyst can be used for seven consecutive runs lacking any substantial loss in its performance.^75^ In comparison to earlier reported methods in the literature, the other highlights of this method are simple handling, lacking any use of additives and external ligands, requiring less quantity of the catalyst for end of reaction, easy and simple partition of the catalyst by an external magnet, reusability of the catalyst for seven successive cycles, and forming high to excellent yields of the products (Scheme 46).

**Scheme 47:**
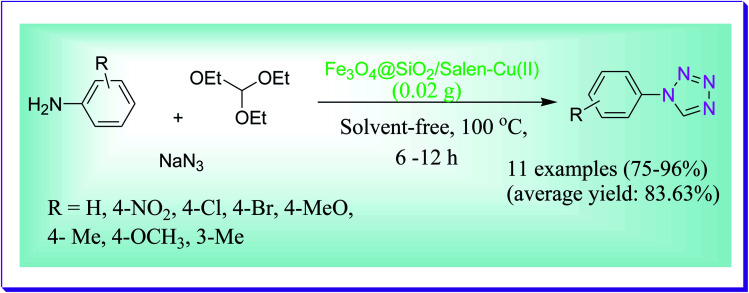
Formation of the tetrazole analogues using Fe_3_O_4_@SiO_2_/salen of the Cu(ii) nanomaterial.

A green three-component reaction was given by Dehghani *et al.* for the preparation of 1- and 5-substituted 1*H*-tetrazoles by nitriles and amines by virtue of magnetite nanoparticle immobilized salen Cu(ii) as a proficient and reusable catalyst under the solvent-free conditions at 100 °C. The nanocatalyst was used for at least 7 successive cycles, lacking any substantial degradation in its catalytic efficiency (Scheme 47).^76^

**Scheme 48:**
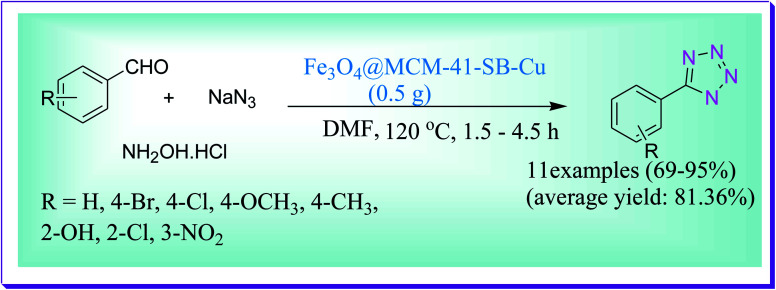
Fe_3_O_4_@MCM-41-SB-Cu nano-catalysed synthesis of 5-substituted-1*H*-tetrazole derivatives.

An efficient one-pot MCR synthesis of 5-substituted-1*H*-tetrazole derivatives was developed by Ahmadi *et al.* through a reaction of aldehydes, hydroxylamine hydrochloride and sodium azide by virtue of the Fe_3_O_4_@MCM-41-SB–Cu nanocatalyst (Scheme 48). The used nanocatalyst due to magnetic nature Fe_3_O_4_@MCM-41-SB-Cu serves simple isolation and is reused numerous times without any reduction in its catalytic efficiency.^77^

**Scheme 49:**
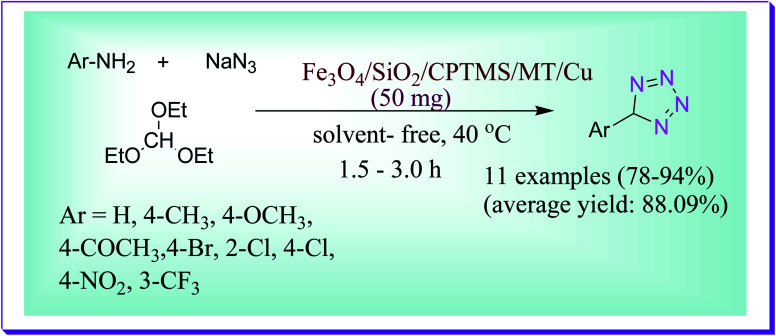
Preparation of 1-substituted tetrazole derivatives catalysed by a copper-based nanocatalyst.

Khorramabadi *et al.* have discussed the preparation of 1-substituted tetrazole derivatives catalysed by the magnetic nanocatalyst Fe_3_O_4_/SiO_2_/CPTMS/MT/Cu at 40 °C through a reaction of aromatic amines, sodium azide and triethyl orthoformate under a solvent-free condition (Scheme 49). By this protocol the desired product was obtained in very excellent yield (94%) and in 2 h reaction time.^78^

**Scheme 50:**
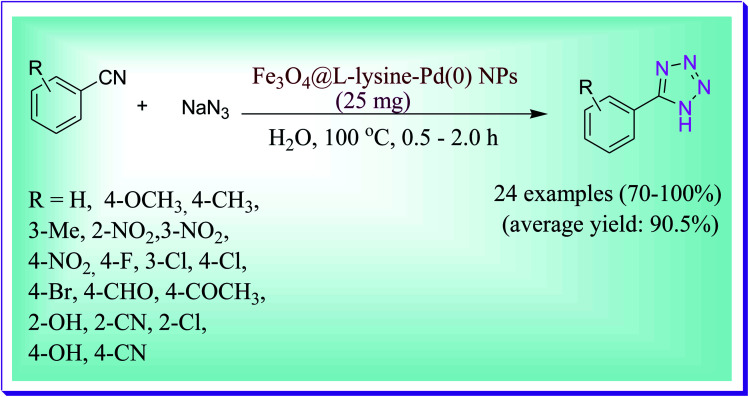
Fe_3_O_4_@l-lysine–Pd(0) NP-catalysed synthesis of 5-substituted 1*H*-tetrazoles.

Muhammad *et al.* reported an efficient and green protocol for the synthesis of 5-substituted 1*H*-tetrazole derivatives by virtue of complex of l-lysine–palladium nanoparticle (NPs) modified Fe_3_O_4_ nanoparticles in aqueous medium *via* the two-component reaction between aryl nitriles and NaN_3_. The current protocol was carried out in a [2+3] cycloaddition manner (Scheme 50). Simple recovery, reusability of Pd NPs, less reaction times, use of a green solvent, excellent yield, operational simplicity, atom and step economy, and simple isolation of heterogeneous catalysts are key features of the present route.^79^

## 7. Conclusion

Heterocyclic motif scaffolds have a wide range of organic and therapeutic applications like hepatoprotective, antidiabetic, geroprotective, vasodilator, bronchodilator, antiatherosclerotic, antitumor and anticancer activities. The use of economical nanocatalysts for the formation of a variety of heterocycles has benefits like less reaction time, low-cost chemical usage, high yield, simple work-up process and very particular reaction. The use of nanocatalysts can also be useful for the formation of diverse heterocycles which are very hard to prepare by traditional protocols. By using an external magnet, magnetically reusable nanocatalysts can be eagerly isolated from reaction medium lacking requirement of centrifugation, filtration or other tedious workup procedures. This review focused on the nanoparticles and their application as magnetically reusable nanocatalysts to choose the most suitable procedure for the synthesis of five-membered N-containing heterocycles. We consider that this review will unlock a new way for the formation of therapeutic and biologically active molecules. We hope to make better successes in this area in the upcoming time.

## Abbreviations

AEPH_2_: 2-Aminoethyl dihydrogen phosphate
CA: Citric acid
CPTES: 3-Chloropropyltriethoxysilane
CPTMS: (3-Chloropropyl)trimethoxysilane
CS: Chitosan
DAD: Diethyl acetylenedicarboxylate
DCM: Dichloromethane
DMF: Dimethylformamide
DMSO: Dimethyl sulfoxide
DTPA: Diethylenetriamine pentaacetic acid
GO: Graphene oxide
HA: Horsetail plant ash
HT: Hydrotalcite
KCC-1: KAUST Catalysis Center
MCM: Mobil composition of matter
MagSilica: Copper nanoparticles dispersed on silica-coated maghemite
M–NiO: Melamine-supported nickel oxide
MPTA-1: Mesoporous poly-triallylamine
PMA: Phosphomolybdic acid
PVA: Polyvinyl alcohol

## Conflicts of interest

There are no conflicts to declare.

## Supplementary Material
